# Hierarchical Dynamics of Ecological Communities: Do Scales of Space and Time Match?

**DOI:** 10.1371/journal.pone.0069174

**Published:** 2013-07-09

**Authors:** David G. Angeler, Emma Göthe, Richard K. Johnson

**Affiliations:** Department of Aquatic Sciences and Assessment, Swedish University of Agricultural Sciences, Uppsala, Sweden; University of San Diego, United States of America

## Abstract

Theory posits that community dynamics organize at distinct hierarchical scales of space and time, and that the spatial and temporal patterns at each scale are commensurate. Here we use time series modeling to investigate fluctuation frequencies of species groups within invertebrate metacommunities in 26 boreal lakes over a 20-year period, and variance partitioning analysis to study whether species groups with different fluctuation patterns show spatial signals that are commensurate with the scale-specific fluctuation patterns identified. We identified two groups of invertebrates representing hierarchically organized temporal dynamics: one species group showed temporal variability at decadal scales (slow patterns of change), whilst another group showed fluctuations at 3 to 5-year intervals (faster change). This pattern was consistently found across all lakes studied. A spatial signal was evident in the slow but not faster-changing species groups. As expected, the spatial signal for the slow-changing group coincided with broad-scale spatial patterns that could be explained with historical biogeography (ecoregion delineation, and dispersal limitation assessed through a dispersal trait analysis). In addition to spatial factors, the slow-changing groups correlated with environmental variables, supporting the conjecture that boreal lakes are undergoing environmental change. Taken together our results suggest that regionally distinct sets of taxa, separated by biogeographical boundaries, responded similarly to broad-scale environmental change. Not only does our approach allow testing theory about hierarchically structured space-time patterns; more generally, it allows assessing the relative role of the ability of communities to track environmental change and dispersal constraints limiting community structure and biodiversity at macroecological scales.

## Introduction

Theory posits that ecological communities consist of species groups that operate in different scaling regimes, wherein the sets of abiotic and biotic organizing variables differ across hierarchical scales [1], [2]. These sets of variables often change abruptly from one hierarchical scale to the next, creating discontinuous or cross-scale structure and non-linear patterns in the communities [3], [4]. Critical to this hierarchical organization, and thus our understanding of ecological and other complex systems, is the duality of processes that operate both in space and time (“space-time duality”). This duality reflects the imprints of processes that act at spatial scales from local to regional to biome and temporal scales ranging from seconds to years to millennia [5]. For example, biological interactions entrain community assembly relatively rapidly at the local scale of habitats, biogeographical processes act over regional spatial and paleoecological temporal scales, and phylogenetic factors are mainly evident over spatially broad domains with slow dynamics [4]. These processes can also self-organize, in the sense that they can produce patterns that reinforce the processes that produced the patterns [6].

Ecologist have developed tools that allow for an assessment of the hierarchical, multiscale structure of ecological systems from either a spatial [7], [8] or temporal perspective [9], [10]. Most spatial studies have the drawback that they provide only single snapshots of community structure across spatial scales that limits an assessment of the dynamic component of the space-time duality. Although a method has been developed to evaluate the stability of abundances through time by assessing space-time interactions [11], temporal patterns at different scales are not explicit in this method. Temporal studies, on the other hand, have supported the theory that the dynamic system structure in terms of speeds of processes varies with scale [5]. For instance, Angeler et al. [12] have used time series modeling to study invertebrate community dynamics during a 20-year period in 26 lakes across Sweden. Consistent with theoretical predictions, one group of invertebrates showed decadal-scale variability associated with climatic variability and regional acid-deposition; that is, environmental factors that operate at regional spatial scales. In contrast, a second species group showed short-term (3–5 year) fluctuation patterns that were unrelated to environmental variables. Notwithstanding, time series modeling has also fallen short of dealing with the space-time duality by not accounting for spatial signals in the scale-specific temporal patterns. Thus neither spatial nor temporal modeling has thus far succeded to analyze the space-time duality of hierarchically organized systems simulatenously and in a coherent way. Identifying relevant scales of space and time influencing scale-specific patterns and processes is a pervasive problem in the ecological sciences [13].

The aim of this paper is to study the space-time duality in the hierarchical organization of communities by assessing spatial signals in the cross-scale structure of time series. More specifically, we test the hypothesis that spatial scales of observations are commensurate with the temporal scales of community dynamics at different hierarchies of ecological organization. That is, broad-scale spatial patterns should match temporal patterns that unfold on broader (e.g. decadal) time scales, and finer-scale spatial patterns should be associated with temporal processes on shorter (e.g. yearly) time scales. We test these conjectures using macroinvertebrate communities in lakes that serve as excellent model systems. First, previous research has shown that community dynamics follow partly theoretical predictions; that is, the temporal dynamics are hierarchically organized with faster and slower dynamics clearly operating in different temporal scaling regimes [12]. Second, lakes have a clear insular metacommunity structure [14], meaning that both their position in the landscape is fixed and their habitat boundaries clearly delineated. This assures that spatial signals in the temporal patterns of community dynamics arise from the dynamics of the invertebrates without being confounded by dynamical habitat changes in the landscape over time. Third, invertebrates are structured by spatial factors at different hierarchical scales [15], [16], which allows testing whether extents of spatial scales matches temporal scales across ecological hierarchies. However, the underlying cause of a spatial signal can vary between hierarchical scales highlighting the need to closer scrutinize potential causes.

Spatial processes on community structure can take a variety of forms, including dispersal limitation and source-sink dynamics or mass effects, species extinctions and evolutionary processes [17], [18]. These factors are not mutually exclusive because they are revealed at different spatial scales, commensurate with the time needed for these factors to manifest [19]. For example, evolutionary processes and extinctions can unfold over centuries, leading to historically contingent, biogeographical patterns in species diversity and community structure [20], [21]. In fact, biogeographical patterns are evident in the community composition and structure of invertebrates in Swedish lakes, coinciding with ecoregions that differ both in vegetation, altitude and climate [22]. Biogeographical patterns, in turn, suggest that communities are dispersal limited at broad spatial scales [23]. However, dispersal limitation is not only manifested at biogeographical scales; it can influence community structure also at much smaller spatial scales such as boreal headwater stream catchments [16]. This highlights that dispersal limitation can emerge from different processes depending on the scale of observation. Because spatial signals in macroinvertebrate metacommunity structure have often been associated with dispersal limitation [15], [16], we infer these processes based on multiple lines of evidence.

We expect that if historical biogeography in the form of ecoregion delineations is the spatial analogue to temporal community change at decadal scales [12], then the spatial signal in the temporal patterns should track broad-scale (i.e. ecoregion) patterns. In contrast, if according to theory shorter-term fluctuations are related to finer scale patterns in space, we expect the spatial structure to reflect, for example, within-ecoregion structures. If the spatial signals arise because the invertebrate communities are dispersal limited across these scales, then the temporal patterns of change should be associated with species with poor dispersal abilities (i.e. overland flight). Thus, in addition to assessing spatial signals across temporal scales qualitatively, we evaluate these signals quantitatively through a dispersal trait analysis [16]. This research will allow us to test the conjecture that spatial and temporal processes are commensurate across hierarchical scales of ecological systems. Because we analyze 20-year time series, a period of documented environmental change across Swedish lakes [24], we will also be able to assess whether the imprints of environmental change manifest distinctly across the temporal scales identified over this period.

## Materials and Methods

### Ethics Statement

All field sampling and laboratory analyses reported in this study are part of the Swedish National Lake Monitoring Program, and are therefore regulated by the Swedish Agency for Marine and Water Management (HaV). All data are made freely available to the public and no permission for use of the data is therefore required. It is also confirmed that the field studies did not involve endangered or protected species.

### Study Area

Twenty-six lakes from the Swedish National Lake Monitoring Program were selected for this study based on longest available time series. These lakes were environmentally heterogeneous, spanning gradients in water clarity (Secchi depth: 1.1 to 10.7 m; water color: <0.01 to 7.98± mg Pt L^−1^), acidity status (pH: 4.6 to 7.3; alkalinity: <0.01 to 0.30 meq L^−1^) and lake size (0.11 to 5.43 km^2^) [12]. Samples for water chemistry and littoral invertebrate assemblages have been collected during the last 20 years (1988–2007) from these lakes. For more information see [25].

### Sampling

Standard sampling and analyses protocols for abiotic and biological variables were used throughout the period of our study. These standard protocols are certified and quality controlled through the Swedish Board for Accreditation and Conformity Assessment (SWEDAC). For this study we used water quality data that were obtained from surface water samples (taken at 0.5 m depth), which were collected once in summer (usually August) and autumn (October) at a mid-lake station in each lake. Previous research has shown that the mid-lake samples are representative for other lake areas [26].

Water was collected with a Plexiglas® sampler and kept cool during transport to the laboratory. Samples were analyzed for electrical conductivity, water temperature, and variables including those indicative of acidity (pH, alkalinity, SO_4_^2−^ concentration), nutrients (Total P, NH_4_-N), and water clarity (Secchi depth transparency, total organic carbon). All physicochemical analyses were done at the Department of Aquatic Sciences and Assessment following international (ISO) or European (EN) standards when available [27]. Measurement intervals and uncertainties for each variable can be found at [28].

Sampling of benthic invertebrates followed Swedish standards throughout the study period (SS-EN 27828). Benthic invertebrates were collected from wind-exposed, vegetation-free littoral habitats in late autumn (October–November) each year. Five samples were taken using standardized kick sampling with a hand net (0.5 mm mesh size). Each sample was taken by disturbing the bottom substratum for 20 seconds along a 1 m long stretch of the littoral region at a depth of c. 0.5 m; thus a total area of 1.25 m^2^ was sampled in each lake. Samples were preserved in 70% ethanol in the field and processed in the laboratory by sorting against a white background with 10× magnification. Invertebrates were sorted, identified to the finest taxonomic unit possible and counted using dissecting and light microscopes by the same person, a trained taxonomist, throughout the study, thereby reducing a researcher-based bias in sample evaluation.

### Statistical Analyses

**Figure 1:**
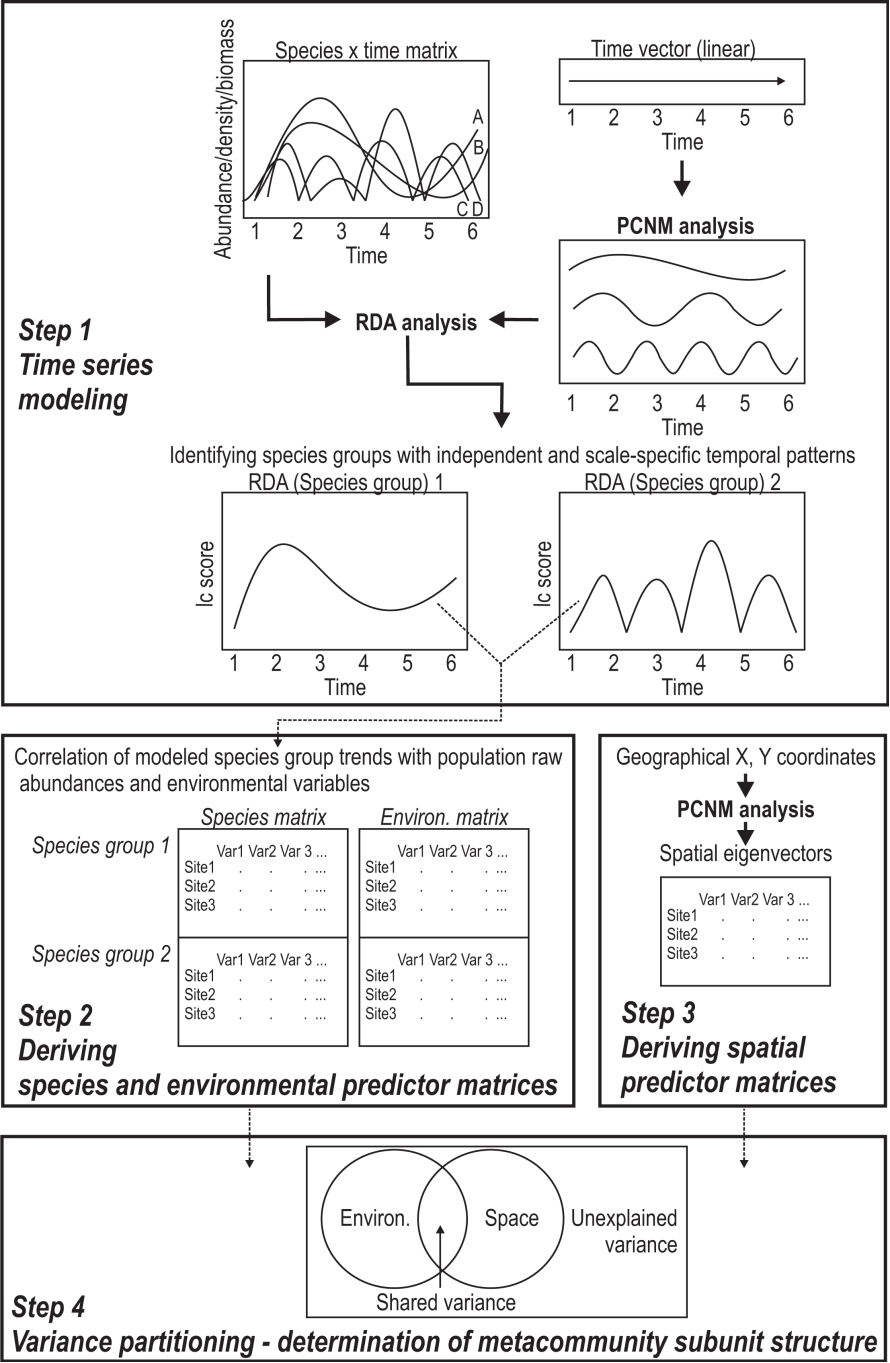
Outline of statistical approaches. Flow chart summarizing the sequence of statistical methods used in this study. Step 1 comprised the time series modeling, based on RDA where time was modeled with a PCNM approach, to determine the independent temporal trends of species groups of invertebrate communities in the lakes. Steps 2 and 3 describe the preparation of environmental and spatial matrices, which were then used in the variance partitioning (step 4). For further details see methods.

Because no unified statistical procedure exists that allows testing the space-time duality of hierarchically organized systems, we used an approach that involves a sequence of independent statistical tests. The steps of this approach are summarized in Figure 1 and involved the determination of variability of invertebrates at different temporal scales (Step 1), the creation of environmental (Steps 2) and spatial matrices (Step 3), and the determination of environmental and spatial factors of community change at each scale using variance partitioning analyses (Step 4).

### Revealing Temporal Variability of Invertebrates at Different Scales (Step 1)

We used a multivariate time series modeling approach, which breaks down the overall temporal structure of communities into independent temporal patterns shown by different groups of species within an assemblage [10]. This approach is based on Redundancy Analysis (RDA) where time is modeled by means of Principal Coordinate of Neighbor Matrices (PCNM). First, the analysis converts a time vector comprised of 20 time steps (i.e. 20 sampling years of invertebrates between 1988 and 2007) into a series of PCNM variables akin to a Fourier transform; that is, a number of different sine waves with different frequencies is obtained through conversion of the time vector (details in [29], [30]). The number of obtained sine waves (or PCNM variables) depends on the length of the time vector that comprises the study period; thus a total of 12 PCNM variables were obtained for the analyses.

Through a forward selection procedure, these PCNMs are related to the community data sets by means of RDA. The RDA retains significant PCNM variables and these are linearly combined in ways to extract temporal patterns from the species matrices; that is, the RDA identifies species with similar temporal patterns in the species × time matrix and uses their temporal pattern to calculate a modeled species group trend for these species based on linearly combined PCNMs. The significance of the temporal patterns of all modeled species groups revealed by the RDA are tested by means of permutation tests.

The RDA relates each modeled species group trend with a significant canonical axis. The R software generates linear combination (lc) score plots, which visually present the modeled temporal patterns of species groups that are associated with each canonical axis. Based on the number of significant canonical axes, the number of modeled species groups with different temporal patterns can be deduced. The ecological relevance of these temporal patterns can be quantified, using adjusted R^2^ values of the canonical axes. The overall temporal structure of the whole community can then be deduced from the number of significant canonical axes in the RDA models.

Because the canonical axes are orthogonal, the PCNM-RDA approach reveals temporal patterns of species groups that are independent from each other. Because temporal trends can be assessed at different scales, this method is useful for more accurately assessing the organization of ecological communities from a complex adaptive systems perspective [12]. This study shows that specific groups of invertebrate species show patterns of decadal change, while other species groups show fluctuation cycles on shorter temporal scales. Thus, rather than assessing change of the “overall” community, we infer patterns of species-group-specific trends that could indicate community organization at different temporal scales.

All relevant steps in the analysis, from conversion of the linear time vector to PCNM variables, to calculation of modeled species group trends to visual presentation of the results in form of lc score plots were carried out with the “quickPCNM” function implemented in R 2.6.0 statistical software package [31]. The calculations, which were based on Hellinger-transformed invertebrate abundance data [32], are therefore based exclusively on an automatic statistical procedure, thereby avoiding potential researcher-induced bias in model construction.

### Identifying Spatial and Environmental Signals of Temporal Variability across Scales

Variance decomposition was used to assess the relative importance of environmental and spatial factors shaping the long-term assembly of species groups. Separate analyses were carried out for each modeled species groups identified across lakes. To account for the temporal structure of each species group explicitly in the analysis, species, environmental and spatial matrices were prepared in the following ways (Steps 2 and 3; Figure 1):

Although variance partitioning analysis is usually done on site by species abundance matrices, we used a modified approach in the present study using correlation coefficients rather than species abundances. Thus the species matrices were created as follows: Spearman rank correlations were first carried out to assess which species correlate significantly with the modeled species group trends for each lake identified through the RDA-PCNM time series modeling. Those species with significant correlations were considered as taxa contributing to temporal change and were used to define the “community composition” of the modeled species groups for each lake. The absolute values of their correlation scores with significant canonical axes from the RDA-PCNM time series modeling were extracted and compiled in species matrices, which were then used in the variance partitioning analysis. Specifically, because time series modeling revealed temporal patterns associated with two canonical axes, we created two species × site matrices to match the spatial analysis with these temporal patterns identified. That is, one species matrix was based on the correlation analysis of the first temporal patterns. It consisted of species as columns and lakes (sites) as rows. The values comprised the correlation coefficients resulting from the correlation between species raw abundances and the modeled temporal scores (linear combination scores) of canonical axis one from the time series models of each lake. The second species matrix was constructed in a similar way but using correlation coefficients derived from the correlations with the second canonical axis. Using the correlation scores associated with significant temporal patterns revealed through time series modeling, rather than time-averaged abundance data of each taxon, better accounts for the explicit temporal structure of species-group change over time in the metacommunity analyses. That is, the correlation scores, but not the time-averaged species abundance, capture the taxa contributions to the modeled temporal changes of species group; because the correlation scores reflect the relative strength of individual taxa contributing to species-group change, structural changes in abundance as a function of modeled group change is accounted for in the analyses.

Regarding the environmental matrix, we followed a similar approach as with the construction of the species matrices. Spearman rank correlations between environmental variables and the modeled species group trends were carried out to identify the set of abiotic variables contributing to species group change through time. Those variables with significant correlations were retained and the absolute values of their Spearman rank correlation coefficients were used as predictor variables in the environmental matrix. To avoid overfitting in posterior variance partitioning analyses, significantly correlated environmental variables were excluded from the matrix. Also here, the correlation coefficients, rather than the time-averaged values of environmental variables, account explicitly for the relative strength of abiotic variables and their relative contribution to species-group change over time, and should therefore better reflect the temporal structure of species-environment associations compared to time-averaged units of environmental variables.

Regarding the spatial matrix, we carried out spatial analyses using the RDA-PCNM procedure outlined above. For these analyses the geographical X (north-south) and Y (east-west) coordinates of each lake composed the explanatory variable matrix, which was then converted into spatial PCNMs using the quickPCNM function. These spatial PCNMs were then related to the matrices of species groups by means of a forward selection in RDA. The spatial PCNMs that were retained in the model, and which therefore explained significant spatial structure in the data sets, were extracted and compiled in a spatial predictor matrix for the variance partitioning analyses.

To determine the relative importance of local environmental variables and spatial (dispersal) processes on the temporal dynamics of species groups, we conducted variation partitioning according to Peres-Neto et al. [33] using the varpart function, implemented in the vegan package, in R [34] (Step 4; Figure 1). Because both the environmental and species group matrices contained many zero values due to insignificant correlations of abiotic variables and individual species with modeled species group trends, the data were Hellinger-transformed prior to the analyses to avoid bias when data with double zero structure are used in ordination methods based on Euclidean distance [32]. The total variation can be partitioned into fractions which comprised of: 1) purely spatial; 2) purely environmental; 3) space uncorrected for environment; 4) environment uncorrected for space; 5) shared variance of space and environment, and 6) residual variance. From these fractions 1), 2) and 6) were the most relevant to our study questions and thus of immediate interest. Variation partitioning was conducted separately for all modeled species groups identified; that is, we associated the community composition of each modeled species group across lakes, revealed through correlation analysis (see above), with environmental and spatial factors.

We classified species according to traits related to flying strength following [35], allowing us to associate the spatial signal with either dispersal limitation or source-sink dynamics which is otherwise not possible in variance partitioning analyses [16]. According to this scheme, taxa can be classified according to the dispersal traits “adult flying strength” (AFS) and “female dispersal” (FD) and assigned to one of two dispersal ability groups: low and high dispersal ability. Low FD are genera that fly <1 km before laying eggs, high FD are genera that can fly >1 km before laying eggs, low AFS are genera that cannot fly into light breeze (weak flyers), and high AFS are genera that can fly into light breeze (strong flyers). Genera that were not included in [35] were excluded from the analysis because of limited knowledge of their dispersal ability (e.g. water mites) [36].

## Results

### Temporal Patterns of Individual Species Groups

**Figure 2:**
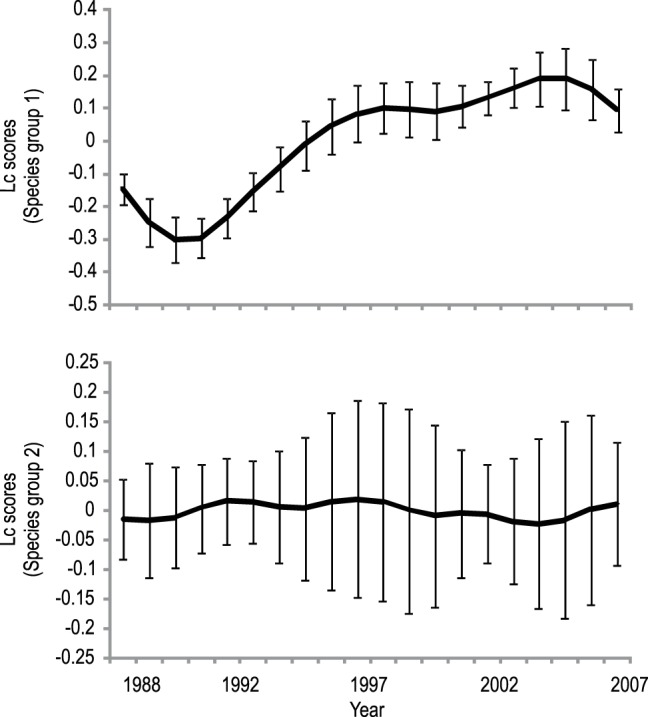
Modeled temporal species group patterns. Temporal patterns of species group 1 associated with RDA axis 1, and species group 2 associated with RDA axis 2 (lc, linear combination, scores) for benthic invertebrates between 1988 and 2007. Shown are the overall patterns (means ± SE) from 26 lakes with significant temporal structure. Both species groups have been identified and separated by the time series modeling analysis based on different temporal structure of the taxa that comprise a community.

The time series analyses using the RDA-PCNM approach detected significant temporal structure associated with different species groups of invertebrates in all of the twenty-six lakes between 1988 and 2007. Significant temporal structure was associated with canonical axes 1 and 2 in the RDA models, indicating the presence of two species groups with independent temporal fluctuation frequencies in the invertebrate communities across all lakes studied. The patterns associated with the first group of species explained on average >50% and the second species group on average <30% of the adjusted variance across all lakes in the models. The temporal structure of the first species group comprised change at broad temporal (decadal) scales (Fig. 2). These patterns cover temporal dynamics that were associated with environmental change over the 20-year study period (see below). We therefore refer to this first temporal frequency group of invertebrates as slowly changing (“slow”) groups that track these slower changes in the environment. By contrast, the second frequency of species groups showed shorter-term periodicity at roughly 5-year intervals, presumably tracking faster ecological processes, and were more variable across lakes compared to the slow patterns, indicated by the standard deviations (Fig. 2); these groups will be referred to as faster-changing (“faster”) groups.

### Environmental Correlates of Group-specific Time Patterns

The importance and strength of environmental correlates of temporal patterns varied with species groups and were evident in the slow rather than the faster species groups across lakes (Table 1). Temporal patterns of the slow group correlated strongly and negatively with sulfate concentrations and electrical conductivity. Correlations of the other environmental variables with the temporal patterns of the slow group were less consistent across lakes. For example, positive correlations between the slow species group and pH and alkalinity were found in a subset of lakes. Subsets of lakes correlated negatively with changes in Secchi disc transparency and positively with total organic carbon. Significant negative correlations between the slow species group patterns and total phosphorus and NH_4_-N were found for only selected lakes. Water temperature was generally a weak correlate of the temporal patterns of the slow group, perhaps because the temporal sampling resolution did not allow us to capture its full variability.

**Table 1:** Spearman rank correlations between water quality variables and species group patterns, revealed by time series modeling, for invertebrate communities between 1988 and 2007.

Lake	SO_4_ (meq L^−1^)	Electr. cond. (mS cm^−1^)	pH	TOC (mg L^−1^)	Secchi depth (m)	TP (µg L^−1^)	Alkalinity (meq L^−1^)	NH_4_-N (µg L^−1^)	Water temp. (°C)
Abiskojaure	ns/0.87***	ns/0.80***	0.58*/ns	ns/ns	ns/ns	−0.69**/ns	ns/0.82***	ns/ns	ns/ns
Älgarydssjön	−0.80***/ns	−0.82***/ns	ns/0.52*	0.55*/ns	ns/ns	ns/−0.6**	ns/ns	0.77***/ns	ns/ns
Allgjuttern	−0.88***/ns	−0.90***/ns	ns/−0.49*	ns/ns	ns/ns 0.70***/0.47*	ns/ns	ns/ns	ns/0.59**	ns/ns
Brännträsket	−0.76***/ns	−0.47*/ns	0.65**/ns	ns/0.56*	ns/ns	ns/ns	ns/ns	ns/ns	0.51*/ns
Brunnsjön	−0.80***/ns	−0.79***/ns	ns/ns	0.57*/ns	−0.68**/ns	ns/ns	ns/ns	ns/0.48*	ns/ns
Bysjön	−0.85***/ns	−0.79***/ns	ns/ns	ns/ns	−0.65**/ns	ns/ns	0.55*/0.46*	ns/ns	ns/ns
Fiolen	−0.82***/ns	−0.81***/ns	0.66**/ns	ns/ns	ns/ns	ns/ns	0.68**/ns	0.55*/−0.46*	ns/ns
Fräcksjön	−0.87***/ns	−0.71***/−0.52*	0.62**/0.47*	0.62**/ns	ns/ns	−0.58**/ns	0.59**/ns	−0.57**/ns	0.58**/ns
Grissjön	−0.80***/ns	−0.74***/ns	ns/ns	0.46*/ns	−0.78***/ns	−0.61**/−0.49*	0.54*/−0.55*	ns/ns	ns/ns
Hagasjön	−0.76***/ns	−0.63**/ns	0.67**/ns	ns/ns	ns/ns	−0.61**/ns	0.56*/ns	ns/−0.57**	ns/ns
Harasjön	−0.88***/ns	−0.83***/ns	ns/ns	ns/ns	−0.72***/ns	ns/ns	ns/ns	0.76***/ns	0.54*/ns
Härsvatten	0.70***/0.59*	ns/ns 0.63**/0.74***	0.70***/−0.46*	ns/ns	ns/ns	−0.52*/ns	−0.77***/ns	ns/0.58**	ns/ns
Humsjön	ns/−0.67**	ns/ns	ns/ns	ns/ns	ns/−0.48*	ns/ns	ns/ns	ns/ns	ns/ns
Jutsajaure	ns/ns	ns/ns	0.51*/ns	ns/ns	ns/ns	ns/ns	ns/ns	ns/ns	ns/ns
Mäsen	−0.86***/ns	−0.63**/ns	0.58*/ns	0.55*/ns	ns/ns	−0.63**/ns	ns/ns	ns/ns	ns/ns
Övre Skärsjön	−0.81***/ns	ns/ns	0.79***/ns	0.54*/ns	ns/ns	ns/ns	ns/ns	ns/ns	0.53*/ns
Remmarsjön	−0.64**/ns	ns/ns	ns/ns	ns/ns	ns/ns	ns/ns	ns/ns	ns/ns	ns/ns
Rotehogstjärnen	−0.77***/ns	−0.76***/ns	ns/ns	0.65**/ns	−0.67**/ns	ns/ns	ns/ns	0.45*/ns	ns/ns
Sännen	−0.79***/ns	−0.78***/ns	0.53*/ns	0.49*/−0.47*	−0.63**/ns	−0.53*/ns	0.65**/ns	ns/ns	ns/ns
Skärgölen	−0.72***/ns	−0.62**/ns	ns/ns	ns/ns	ns/ns	ns/ns	ns/0.68**	ns/ns	ns/ns
Stensjön	ns/ns	−0.53*/0.58**	ns/ns	ns/ns	ns/ns	−0.67**/ns	ns/ns	ns/ns	ns/ns
Storasjö	−0.86***/ns	−0.91***/ns	ns/ns	0.52*/ns	ns/ns	ns/0.57**	ns/ns	ns/ns	ns/ns
Stora Skärsjön	−0.89***/ns	−0.86***/ns	ns/ns	ns/ns	−0.53*/ns	−0.54*/ns	0.71***/ns	−0.59**/ns	0.61**/ns
Stora Envättern	−0.79***/ns	−0.65**/−0.61**	ns/ns	0.49*/ns	−0.57*/ns	ns/ns	ns/ns	0.49*/0.56**	ns/ns
StorTjulträsket	ns/ns	ns/ns	ns/ns	ns/ns	ns/ns	ns/ns	ns/ns	ns/ns	ns/ns
Tväringen	ns/ns 0.57**/0.57**	−0.59**/ns	ns/ns	ns/ns	ns/ns	ns/ns	ns/0.57**	ns/ns	ns/ns

Shown are Spearman rank correlation coefficients (rho) and significance levels (* P<0.05; ** P<0.01; *** P<0.001) for species group 1/species group 2. ns, no significant correlation. Absolute values of rho were used as abiotic variables in the environmental predictor matrices for the variance partitioning analyses of species groups 1 and 2, respectively. Only variables with significant correlations have been included in the table. The table has been modified from [45].

### Taxonomic Composition of Species Groups

Spearman rank correlation analyses revealed that the number of taxa explaining the modeled temporal frequency patterns ranged 3–40 (slow groups) and 2–17 (faster groups). Many invertebrate taxa (e.g., *Caenis luctuosa, Caenis* spp., *Cloeon dipterum, Cloeon* spp. (Ephemeroptera), *Athripsodes bilineatus, Oecetis* sp. (Trichoptera), *Capnia artra* (Plecoptera), *Cladotanytarsus* sp., *Psectrocladius* sp., *Conchapelopia* sp., *Tanytarsus* sp. (Diptera), *Oulimnius* sp. (Coleoptera), *Helobdella stagnalis* (Annelida), Hydracarina, Ceratopogonidae) were found in the slow and faster species groups, but their strength and the significance of their contribution to species group patterns varied among lakes.

### Spatial and Environmental Correlates of Species Group Patterns

**Figure 3:**
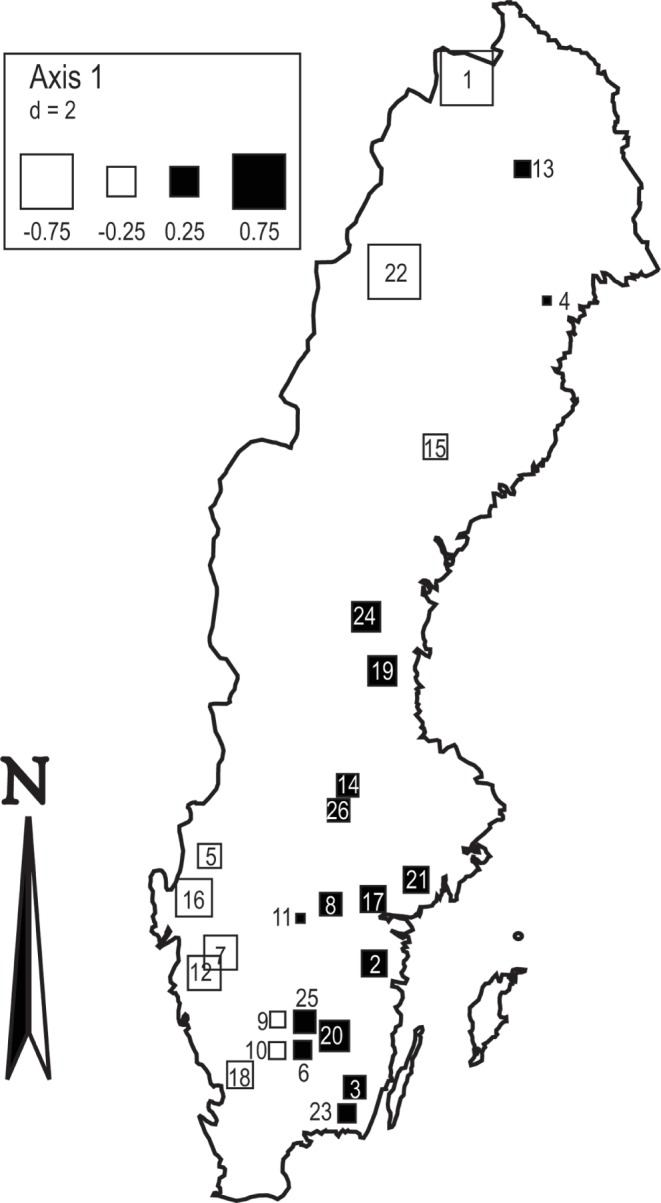
Spatial patterns of temporal patterns. Result from spatial analysis showing broad-scale spatial structure of invertebrates in the time-explicit metacommunity analysis. The size of the symbols are proportional to the lower-order PCNM vectors that describe this spatial structure and the color represents the sign (white = negative, black = positive). Numbers within squares refer to lake identity: 1, Abiskojaure; 2, Allgjuttern, 3, Brunnsjön; 4, Brännträsket; 5, Bysjön; 6, Fiolen; 7, Fräcksjön; 8, Grissjön; 9, Hagasjön; 10, Harasjön; 11, Humsjön; 12, Härsvatten; 13, Jutsajaure; 14, Mäsen; 15, Remmarsjön; 16, Rotehogstjärnen; 17, Skärgölen; 18, Stora Skärsjön; 19, Stensjön; 20, Storasjö; 21, Stora Envättern; 22, Stor-Tjulträsket; 23, Sännen; 24, Tväringen; 25, Älgarydssjön; 26, Övre Skärsjön.

**Figure 4:**
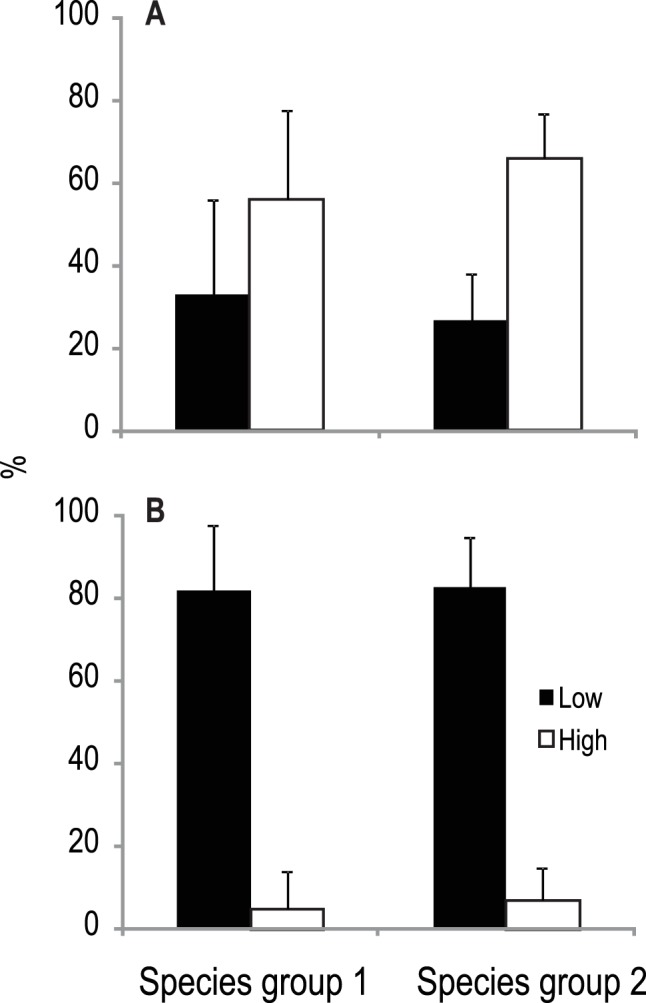
Dispersal trait analysis. Dispersal characteristics (A, female dispersal; B, adult dispersal) of taxa contributing to the temporal dynamics of species groups 1 and 2, respectively. Shown are the mean percentages ± standard deviations of species showing the traits across the study lakes.

The relative importance of environmental and spatial factors correlating with temporal species group change varied between species groups (Table 2). For the slow groups, both pure environmental (adj. R^2^ = 0.006; P<0.001) and spatial factors (adj. R^2^ = 0.016; P<0.001) were significant in the variance partitioning; however, the variance explained by these fractions was generally low. The spatial fraction captured the importance of broad-scale spatial processes, whereby the sets of species contributing to species group change differed along an east-west geographical gradient (Fig. 3). The midge *Tanypodinae* (correlation score with RDA axis 1: −0.94), the stonefly *Capnia atra* (−0.52) and the mayfly *Metretopus borealis* (−0.45) correlated with the temporal change of the slow groups in lakes towards the west (lakes with white squares in Fig. 3), while those in eastern lakes where associated with the isopod *Asellus aquaticus* (0.54), the mayfly *Leptophlebia vespertina* (0.52), and the mollusc *Pisidium* sp. (0.52) (lakes with black squares in Fig. 4). The residual variation that cannot be attributed to environmental and spatial factors was high for the slow species groups (>0.9). The temporal patterns found for the faster species groups were neither explained by environmental nor spatial factors (Table 2).

**Table 2:** Results from variance partitioning analyses.

	Species group 1		Species group 2	
Fractions	adj. R^2^	P	adj. R^2^	P
Environment uncorrected for space	0.010	0.246	0.007	0.313
Space uncorrected for environment	0.062	0.001	0.010	0.280
Pure environment	0.006	0.001	0.002	0.217
Pure space	0.016	0.001	0.001	0.234
Shared variance	0.046		0.001	
Residual	0.945		0.992	

Shown are the variance and significance levels by different fractions explaining the patterns of temporal change of two species groups of invertebrates, identified through the RDA-PCNM time series modeling, with contrasting temporal patterns. Note: no significance levels are calculated for shared and residual variance in the models.

### Dispersal Characteristics of Taxa in Species Groups

Despite different taxa explaining the slow and faster species groups across lakes, dispersal characteristics of these taxa were similar both between species groups and across lakes within each species group (indicated by the relatively low standard deviation) (Fig. 4). Approximately 60% of taxa explaining the temporal patterns of the slow and faster species groups consisted of females that fly >1 km before depositing their eggs, while ca. 40% fly <1 km (Fig. 4A). Overall, however, adult dispersal strength was low for both species groups with >80% of the taxa being weak flyers relative to the <10% that were strong flyers (Fig. 4B). No significant differences were detected when each dispersal class was compared between the slower and faster species groups (one-way ANOVA on arcsine square-root-transformed data: p>0.05).

## Discussion

Despite theory predicting that community dynamics are commensurate in space and time depending on the scales of ecological hierarchies at which these dynamics are explored [4],[5], empirical testing of this space-time duality has so far been limited because of the lack of adequate time series with enough spatial and temporal resolution and statistical methods suitable for identifying cross-scale structure and hierarchically organized dynamics. Using 20 years of monitoring data of lakes situated at a broad spatial gradient in Sweden, we not only identified temporal dynamics in the form of slow and faster species groups across lakes that were consistent with theory, but also that when a spatial signal was detected in the time series the spatial pattern was commensurate with the temporal frequencies observed. That is, the patterns of change of the slow species groups of invertebrates were associated with a broad-scale spatial pattern. What does this spatial signal mean?

Researchers have often invoked historically contingent effects to describe species diversity in plant and animal communities at broad spatial scales [20], [21]. More specifically, evolutionary processes, extinctions and dispersal limitation have been described as biogeographical factors constraining species diversity and community structure in the absence of environmental pressures [18] or when biological adaptations to environmental change is lagging [23]. Evidence exists that the spatial signal in our study results from effects related to historical biogeography. For instance, a relatively good correspondence was found between ecoregion delineations and macroinvertebrate assemblages of the lake littoral zones in Sweden [22]. Ecoregions in Sweden can be delineated mainly based on altitude, vegetation characteristics and climatic factors. In northern latitudes a western high-elevation (arctic-alpine) ecoregion can be differentiated from an eastern low-altitudinal (boreal) ecoregion. Not only did the spatial signal in our analysis correspond well with the delineation between these ecoregion, also species that have been shown to typify these ecoregions (*Capnia atra* in the arctic-alpine ecoregion and *Asellus aquaticus* in the boreal ecoregion; [22]) contributed to the spatial pattern identified in our analysis. Southern Sweden comprises the boreonemoral and nemoral ecoregion with warmer climates and deciduous forests as the dominant vegetation type.

While the relatively good correspondence of the spatial signal with ecoregion patterns in this study supports the interpretation of historical biogeography influencing community dynamics, we acknowledge that the determination of underlying causes is difficult [44]. More specifically, spatial signals have been associated with diametrically opposing processes: dispersal limitation and mass effects [17], but these processes cannot be discerned in variance partitioning studies [37], [38]. Mass effects explaining the spatial patterns at the biogeographical scale of our study would be counterintuitive, so we ascertained by means of a dispersal trait analysis of the invertebrates that the underlying process was dispersal limitation.

The dispersal traits analysis of the taxa that explained the slow species groups of invertebrates revealed that, despite the species groups being dominated by females that can fly>than 1 km before laying eggs, >80% of the taxa were weak flyers at the adult stage [35]. We acknowledge that our characterization of dispersal characteristics is relatively coarse because our current knowledge of invertebrate dispersal traits is still limited [36]. However, it is unlikely that the dominance of the slower groups of invertebrate species with females that disperse more than 1 km indicates mass effects at the macroecological scale of our study. Even strong flyers have been shown to be dispersal limited in small boreal catchments [16], a finding that is supported by some studies that have shown genetic differentiation within and between catchments that could reflect dispersal limitation [39]–[41]. Also, most taxa, especially those that were associated with the spatial signal in the variance partitioning (*Capnia artra, Leptophlebia vespertina, Metretopus borealis*) are either weak flyers [35] or they lack active overland dispersal traits (*Asellus aquatics, Pisidium* sp.). Taken together, these results suggest that dispersal between lakes across the observed west-east gradient is limited and that the spatial signal resulting from the spatial patterns of our study is most likely due to dispersal limitation. This interpretation is in agreement with an increasing body of evidence that dispersal limitation due to biogeographically defined boundaries rather than mass effects (source-sink dynamics) between macroecological spatial units describes historical community assembly of metazoans [23], [42], [43].

We expected to observe a spatial signal in the form of a finer-scale spatial pattern also in the faster species groups but the variance partitioning analysis failed to detect such an effect. Our hypothesis and underlying theory are therefore only partly supported. However, we acknowledge that this may be due to limitations with our approach. The temporal patterns of the faster species group showed fluctuation cycles on much shorter (roughly 5 years) time spans that explained less variance in the time series models compared to the slower species groups. We assume that while time series modeling was sensitive enough to identify distinct temporal patterns in the invertebrates, the sampling resolution for the faster species groups might have been either too coarse or the identified patterns too weak for detecting a significant spatial signal for these species groups. Also, the temporal patterns of the faster species groups were more variable across the lakes compared to the slower groups, suggesting that the faster group may be driven more by system intrinsic factors relative to an extrinsic control of the slower species group [12]. Weak patterns and high variability are, however, a common problem in studies based on survey data. These can often have a high residual variation due to the accumulation of noise related to sampling, ecosystem history and intrinsic variability [44]. We also took a conservative approach by correcting R^2^-values by the number of explanatory variables [33], and the use of a sequence of statistical procedures for assessing the hierarchical space-time duality may have further introduced noise, decreasing the variance explained in the models. Consequently, the variance explained is lower compared to other variance partitioning studies [45], [46]. Importantly, however, the main conclusions of our study are based on the formal analysis of patterns of R^2^-values across species groups rather than on the actual values of these estimates.

We conclude by highlighting two aspects of our study related to methodology and inference. Regarding the former, our approach was based on the PCNM methodology, which has identified patterns of hierarchically structured community dynamics. Although, refinements of the original PCNM procedure in spatial or time series modeling using canonical ordination have been suggested [7], [8], the PCNM method was powerful enough to identify patterns that were both credible and consistent with theoretical predictions [4], [5]. We therefore regard the PCNM approach suitable for inference making in the present study.

Regarding inference, our primary interest was to test the space-time duality of ecological patterns across hierarchical scales. However, our results allowed for inferences beyond this goal. Having included both spatial and environmental factors in the analysis provided opportunities to assess the relative importance of both factors in long-term community change. We found that not only space but also environmental variables were associated with the temporal dynamics of the slow group of invertebrates. This scale-specific imprint of environmental variables has also been found in previous studies and was due to broad-scale processes related to reduced acid deposition and climatic variability [12]. These patterns were coherent across Swedish lakes suggesting that the patterns of temporal change were not contingent on the biogeographical patterns associated with ecoregions in Sweden [47]. Previous studies have shown that estimates of the relative importance of partial space or environmental components may fail to accurately represent environmental and spatial components of community variation [48], [49], limiting inference about the role of niche-based or neutral processes in community assembly using variance partitioning analysis. We do not expect such a problem in our analysis because the environmental and spatial effects have been assessed at different temporal scales. That is, we accounted explicitly for time in the analysis covering a dynamic temporal component of environment and species over the 20-year study period. By contrast, space comprised a static variable. These different temporal windows of measurement reduce the risk that environmental and spatial predictors confound each other in the analysis. Based on previous studies that temporal trends were synchronized across Swedish lakes and the results from the present study, we conclude that regionally distinct sets of taxa responded similarly to broad-scale environmental change. More generally, our results show how an assessment of the hierarchically structured space-time duality can contribute to the debate of the relative role of the ability of communities to track environmental change (niche-based processes) versus dispersal constraints (neutral processes) limiting community structure and biodiversity at macroecological scales.
